# The relationship between cardiac injury, inflammation and coagulation in predicting COVID-19 outcome

**DOI:** 10.1038/s41598-021-85646-z

**Published:** 2021-03-22

**Authors:** Alessandro Mengozzi, Georgios Georgiopoulos, Marco Falcone, Giusy Tiseo, Nicola Riccardo Pugliese, Meletios A. Dimopoulos, Lorenzo Ghiadoni, Greta Barbieri, Francesco Forfori, Laura Carrozzi, Massimo Santini, Fabio Monzani, Salvatore De Marco, Francesco Menichetti, Agostino Virdis, Stefano Masi, Pisa Covid Study Group, Agostini o Degl’Innocenti Sabrina, Biancalana Martina, Borselli Matteo, Nencini Elia, Spinelli Stefano, Antognoli Rachele, Calsolario Valeria, Paterni Simone, Baldassarri Rubia, Bertini Pietro, Brizzi Giulia, Corradi Francesco, Della Rocca Alessandra, Guarracino Fabio, Malacarne Paolo, Monfroni Marco, Piagnani Chiara, Celi Alessandro, Cinotti Francesco, Park Naria, Cipriano Alessandro, Colangelo Luciano, Sonato Chiara, Galfo Valentina, Monica Uliana, Ruberti Francesca, Forotti Giovanna, Sciuto Maria

**Affiliations:** 1 Department of Clinical and Experimental Medicine, University of Pisa, Pisa, Italy; 2 School of Biomedical Engineering and Imaging Sciences, King’s College London, London, UK; 3 Department of Clinical Therapeutics, School of Medicine, National and Kapodistrian University of Athens, Athens, Greece; 4 Department of Anaesthesia and Intensive Care, University Hospital of Pisa, Pisa, Italy; 5 Department of Cardiothoracic and Vascular Department, University of Pisa, Pisa, Italy; 6 Department of Emergency Medicine, Azienda Ospedaliera Universitaria Pisana, Pisa, Italy; 7 Fifth Medical Unit, Azienda Ospedaliera Universitaria Pisana, Pisa, Italy; 8 Institute of Cardiovascular Science, University College London, London, UK; 9 Geriatrics Unit, Azienda Ospedaliera Universitaria Pisana, Pisa, Italy; 10 Infectious Diseases Unit, Azienda Ospedaliera Universitaria Pisana, Pisa, Italy; 11 Fourth Medical Unit, Azienda Ospedaliera Universitaria Pisana, Pisa, Italy

**Keywords:** Predictive markers, Prognostic markers

## Abstract

High sensitivity troponin T (hsTnT) is a strong predictor of adverse outcome during SARS-CoV-2 infection. However, its determinants remain partially unknown. We aimed to assess the relationship between severity of inflammatory response/coagulation abnormalities and hsTnT in Coronavirus Disease 2019 (COVID-19). We then explored the relevance of these pathways in defining mortality and complications risk and the potential effects of the treatments to attenuate such risk. In this single-center, prospective, observational study we enrolled 266 consecutive patients hospitalized for SARS-CoV-2 pneumonia. Primary endpoint was in-hospital COVID-19 mortality. hsTnT, even after adjustment for confounders, was associated with mortality. D-dimer and CRP presented stronger associations with hsTnT than PaO_2_. Changes of hsTnT, D-dimer and CRP were related; but only D-dimer was associated with mortality. Moreover, low molecular weight heparin showed attenuation of the mortality in the whole population, particularly in subjects with higher hsTnT. D-dimer possessed a strong relationship with hsTnT and mortality. Anticoagulation treatment showed greater benefits with regard to mortality. These findings suggest a major role of SARS-CoV-2 coagulopathy in hsTnT elevation and its related mortality in COVID-19. A better understanding of the mechanisms related to COVID-19 might pave the way to therapy tailoring in these high-risk individuals.

## Introduction

Elevated high sensitivity troponin T (hsTnT) at admission, a well-established marker of cardiac injury, has been shown to be common and highly predictive of adverse outcome in patients with Coronavirus Disease 2019 (COVID-19)^1–3^. The mechanisms underlying to troponin elevation in COVID-19 are thought to be multifactorial. Hypoxia related to interstitial pneumonia potentially leading to non-ischemic myocardial injury, as well as the exaggerated immune-inflammatory response, marked by an elevation of the C-reactive protein (CRP), are amongst the two most commonly cited mechanisms potentially leading to hsTnT elevation in these patients^4,5^.

More recently, activation of coagulation pathways has been described in a substantial proportion of patients with severe COVID-19 and their presence has been related to the development of multi-organ dysfunction, resulting in an increased risk of death^6^. An hyperactivation of the coagulation system, marked by an elevation of the D-dimer, has been associated to an increased risk of mortality in COVID-19^3,7^. *Post-mortem* studies have confirmed the presence of a COVID-19-related coagulopathy characterized by the development of thrombotic microangiopathy in multiple organs, including the heart vessels^8^. Such anatomical alterations can contribute to the elevation of hsTnT independently from the severity of the inflammatory response, as they can further aggravate the delivery of an adequate oxygen supply to the myocardium. The importance of control the coagulatory homeostasis in COVID-19 is recently under attention as some studies have reported a beneficial effect of anticoagulation therapies in these patients^7,9,10^. However, beyond the large number of reports describing associations between hsTnT with mortality in COVID-19, no studies have comprehensively explored the relative contribution of coagulopathy, hypoxia and inflammation to the increase in hsTnT during SARS-CoV2 pneumonia, nor the importance of these pathways in defining the risk of mortality in COVID-19.

A better understanding of the plethora of ischemic and non-ischemic causes of myocardial injury related to COVID-19 might inform on the most appropriate treatment to be used in patients presenting with elevated levels of hsTnT.

In this study, we evaluated the relative importance of the hypoxia, inflammatory response and activated coagulation in the elevation of the hsTnT levels in patients with a diagnosis of Severe Acute Respiratory Syndrome Coronavirus 2 (SARS-CoV-2).

We also assessed the complex interaction between hsTnT, D-dimer and C-reactive protein (CRP) in defining the risk of an adverse outcome or hospital complications in the same population. Finally, given the potential contribution of inflammation and coagulation abnormalities to the myocardial injury in COVID-19, we assessed the capacity of immunomodulating drugs or low molecular weight heparin to modify the relationship between high hsTnT levels and increased risk of mortality.

## Methods

### Population and data collection

This single-center, prospective, observational study included 266 consecutive patients hospitalized for SARS-CoV-2 pneumonia at the tertiary care University hospital of Pisa, Tuscany, Italy, between 4th March and 31st March 2020. All patients had a diagnosis of SARS-CoV-2 infection confirmed by positive results of PCR testing of a nasopharyngeal swab. People with high clinical and radiological suspicion of SARS-CoV-2 infection were tested at least twice (at admission and after 12 to 24 h) and infection was excluded only after at least two negative nasopharyngeal swab or two negative nasopharyngeal swab and a negative bronchoalveolar lavage testing, depending on the clinical suspicion. Pneumonia was established with a plain chest CT reviewed by trained radiologists. Demographic, clinical, laboratory, instrumental, treatment, and outcome data were collected as previously reported^11^; hsTnT upper reference limit was 14 ng/L. Venous and arterial blood samples for standard biochemistry and arterial blood gas analysis were collected at the time of hospitalization and repeated according to clinical practice and depending on the patient clinical conditions. The study complied with the edicts of the 1975 Declaration of Helsinki and. All experimental protocols were approved by the institutional ethic committee Comitato Etico Area Vasta Nord Ovest (CEAVNO).

### Outcomes

The primary endpoint of the study was considered the in-hospital mortality. Secondary endpoints were considered the admission to Intensive Care Unit (ICU), the development of acute respiratory distress syndrome (ARDS, according to the Berlin Definition)^12^ and the need for respiratory support by invasive or non-invasive mechanical ventilation.

### Statistics

Continuous variables are presented as mean ± standard deviation or median (interquartile range) for variables not following normal distribution. Nominal variables are summarized as absolute counts and percentage values (%). Normal distribution of all continuous variables was graphically assessed by histograms and quantile–quantile plots. Differences in variables of interest between pre-defined groups (*i.e.* according to sex, history of CVD, increased admission levels of hsTnT) were examined with the independent’s samples Student’s T Test or the non-parametric Mann–Whitney test and the chi-squared test for continuous and nominal variables, respectively. To identify independent determinants of admission hsTnT concentrations, we used multivariable linear regression models. Subsequently, we employed Cox proportional-hazards models to evaluate the association between admission variables and the occurrence of the primary endpoint (all-cause mortality). We used the Schoenfeld residuals to test the proportional hazards assumption. Kaplan Meier curves were plotted to illustrate the difference in survival probabilities (by implementing the Log rank test) according to admission hsTnT levels. Respectively, we used multivariable logistic regression analysis to appraise the association of hsTnT with secondary outcomes, including incidence of ARDS, invasive and/or non-invasive mechanical ventilation and transfer to ICU. Finally, we utilized two-level linear mixed models with random effects (random intercept and random coefficient) and unstructured variance–covariance matrix to assess whether longitudinal changes in hsTnT levels were associated with fluctuations in levels of CRP and D-dimer across the hospitalization. In all multivariable regression models (Cox regression, logistic regression and linear mixed models), we controlled for a pre-specified set of biologically plausible covariates, including age, gender, history of CVD and creatinine levels. These were selected given their well-established association with the COVID-19 mortality risk reported in several previous reports^13,14^. In linear regression and linear mixed models, we ensured the normal distribution of the dependent outcome by transforming continuous hsTnT with the natural logarithm. Associations are presented as Hazard Ratio (HR) or Odds Ratio (OR) or coefficients with 95% confidence intervals (CI).

In this study, we included all consecutive patients with COVID-19 admitted to the Hospital of Pisa, Italy, until the 31st March 2020. In this context, no formal power analysis was performed. Still, we sought to retain a ratio of 5 to 10 events or observations per covariate used in Cox or logistic regression and linear regression models. In Cox regression models with a large number of covariates (i.e. predictors of mortality among different treatments after adjustment for hsTnT, age, gender, history of CVD and creatinine levels), we performed bootstrapping with 200 replicates and derived corrected confidence intervals.

Statistical analysis was conducted with STATA package, version 12.1 (StataCorp, College Station, Texas USA). All tests were 2-tailed. We deemed statistical significance at *P* < 0.05.

### Ethics approval

The study complied with the edicts of the 1975 Declaration of Helsinki. All experimental protocols were approved by Comitato Etico Area Vasta Nord Ovest (CEAVNO).

###  Consent to participate

 Informed consent was obtained from all individual participants or their legal guardians included in the study.

### Consent to publication

Informed consent for data publications was obtained from all individual participants or their legal guardians included in the study.

## Results

### Prognostic role of hsTnT

**Figure 1:**
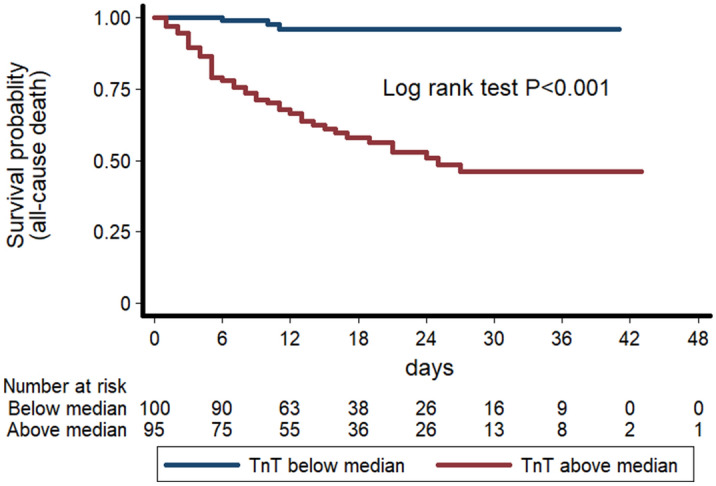
Kaplan–Meier survival curves for all-cause death according to median levels of admission hsTnT levels (blue line = below median; red line = above median).

The baseline clinical characteristics of the population stratified by mortality status at follow up are reported in Table 1. Mortality rate was high (32%) at the end of the follow-up. People who died were older and presented a higher blood pressure, prevalence of male sex, CVD comorbidities and chronic liver and kidney diseases. Their lymphocyte and platelet counts were lower, while they showed an elevated AST, creatinine, CRP, D-dimer and creatine kinase. Lower PaO_2_ and PaO_2_/FiO_2_ on admission were also detected in patients who died during hospitalization. Remarkably, hsTnT was the parameter with the greatest difference between the group of patients who died during admission compared to subjects who survived (fivefold difference). In Cox regression models, hsTnT levels were strongly associated with the risk of in-hospital mortality, even after adjustment for age, sex, previous history of CVD, creatinine levels (Table 2 and Fig. 1). Addition of the PaO_2_/FiO_2_ to the model did not attenuate the significant association of hsTnT with mortality (HR = 2.18 per 1 SD increase, 95% CI 1.36–3.5, *P* = 0.001). Weaker o no associations were observed between hsTnT and risk of ARDS, ICU admission and ventilation. The association between hsTnT and mortality was significant in both sexes, as well as in people with and without previous history of CVD. Only age modified the relationship between hsTnT and risk of mortality, so that in subjects > 75 years old this relationship was stronger than in those < 75 years old (HR = 1.012, *P* = 0.005 vs HR = 1.001, *P* = 0.051).

**Table 1:** Differences in clinical characteristics at admission according to survival status.

Variable	N	In-hospital mortality		*P*-Value
		Survived (N = 202)	Died (N = 64)	
Age, years	266	63 (15)	79.3 (10)	< 0.001
Male sex, n (%)	266	130 (64.4)	50 (78.1)	0.040
Current smoking, %	243	9 (5)	3 (5)	0.925
Systolic blood pressure, mmHg	240	125 (22)	133 (20)	0.011
Diastolic blood pressure, mmHg	240	71 (15)	76 (13)	0.011
Heart rate, bpm	242	90 (77–110)	85 (75–95)	0.057
PaO_2_*	240	71 (60–86)	59 (41–73)	< 0.001
PaO_2_/FiO_2_*	254	290 (233–362)	200 (150–276)	< 0.001
Glucose, mg/dL	252	132 (59)	145 (51)	0.145
Haemoglobin, g/dl*	258	13.5 (12–14.7)	13 (11.6 -14.2)	0.467
Leukocyte count, cells/μl*	259	6580 (4960–9140)	6640 (5150–8940)	0.860
Lymphocyte count, cells/μl*	257	1000 (680–1317)	750 (508–1110)	0.001
Neutrophils count, cells/μl*	256	4700 (3220–6940)	5700 (3790–7320)	0.164
Platelets count, cells/μl*	259	195 (149–247)	172 (125–215)	0.021
INR*	248	1.22 (1.15–1.32)	1.23 (1.17–1.50)	0.183
Total Bilirubin, mg/dL*	249	0.50 (0.36–0.74)	0.55 (0.38–0.78)	0.509
Aspartate transaminase, U/L*	237	32 (23–46)	39 (26.5–54.5)	0.025
Alanine transaminase, U/L*	253	27 (19–48)	25 (16–45)	0.327
Creatinine, mg/dL*	255	0.94 (0.78–1.14)	1.3 (1.05–1.91)	< 0.001
Sodium, mEq/L*	258	138 (136–140)	138 (135–142)	0.565
Potassium, mEq/L*	253	3.98 (3.73–4.28)	4.15 (3.78–4.64)	0.011
D-dimer, mg/L*	84	0.45 (0.22–1.04)	0.74 (0.54–2.86)	0.043
Lactate Dehydrogenase, U/L*	139	304 (216–427)	385 (255–502)	0.077
Creatine phosphokinase, U/L*	119	85 (48–166)	218 (80–465)	0.012
High sensitivity troponin T, ng/L*	200	12 (7–23)	59 (33–119)	< 0.001
Procalcitonin, ng/mL*	222	0.11 (0.06–0.26)	0.21 (0.12–0.69)	< 0.001
C-reactive protein, mg/dl*	247	6.26 (2.60–13.60)	8.68 (5.36–17.23)	0.008
**Comorbidities**				
Chronic obstructive pulmonary disease, n (%)	266	17 (8.42)	19 (29.69)	< 0.001
Asthma, n (%)	265	16 (7.92)	2 (3.17)	0.191
Diabetes mellitus, n (%)	266	36 (17.82)	15 (23.44)	0.320
Arterial hypertension, n (%)	266	81 (40.10)	41 (64.06)	< 0.001
History of cardiovascular disease, n (%)	266	52 (25.74)	34 (53.13)	< 0.001
History of cerebrovascular disease, n (%)	266	14 (7)	13 (20)	0.002
Diagnosis of dementia, n (%)	266	13 (6)	7 (11)	0.234
Diagnosis of Cancer, n (%)	266	28 (14)	11 (17)	0.512
Haematological malignancy, n (%)	266	3 (1.49)	3 (4.69)	0.133
Chronic kidney disease, n (%)	266	11 (5)	12 (19)	0.001
Dialysis, n (%)	266	3 (1)	2 (3)	0.400
Chronic liver disease, n (%)	266	4 (2)	7 (11)	0.002

*P*-values are derived from independent samples Student’s T Test or the Mann Whitney test for continuous variables and the chi squared test for categorical variables; *Median and interquartile range.

**Table 2:** HRs for each outcome for 1 SD increase in hsTnT levels.

Outcome	HR	95% CI	*P*-value	
**All cause death**				
Unadjusted	1.2	1.07–1.34	0.002	
Adjusted	2.28	1.41–3.68	0.001	
**ARDS**				
Unadjusted	2.12	0.824–5.45	0.119	
Adjusted	1.58	0.659–3.8	0.305	
**ICU admission**				
Unadjusted	1.11	0.838–1.48	0.462	
Adjusted	1.19	0.886–1.6	0.219	
Ventilatory support*				
Unadjusted	1.19	0.863–1.64	0.289	
Adjusted	1.01	0.532–1.91	0.983	

Adjusted associations are controlled for the effect of age, gender, cardiovascular disease and admission creatinine. *Ventilatory support = requirement for invasive and non-invasive ventilation.

Similar to hsTnT, D-dimer was associated with the risk of in-hospital mortality at Cox regression analysis, while weaker or no associations were identified with the other outcomes (Table 3). The association between D-dimer and mortality risk remained significant even after adjustment for age, sex, previous history of CVD and levels of creatinine (HR = 1.11, 95% CI 1.03–1.185, *P* = 0.005). Conversely, CRP was not associated with an increased mortality risk, although it was related with the risk of ARDS, ICU admission and requirement of respiratory ventilation (Table 3).

**Table 3:** Unadjusted and multi-adjusted associations of 1 SD increase of C-reactive protein and DD with the main study outcomes.

Outcome	CRP				D-dimer			
	Univariable		Multivariable		Univariable		Multivariable	
	HR/OR 95% CI	*P*-value	HR/OR 95% CI	*P*-value	HR/OR 95% CI	*P*-value	HR/OR 95% CI	*P*-value
Death*	1.01(0.993–1.02)	0.337	1.026(.996–1.057)	0.095	1.09 (1.03–1.15)	0.001	1.11(1.03–1.185)	0.005
ARDS^¥^	1.03 (1.003–1.06)	0.032	1.026 (0.995–1.06)	0.107	1.32 (0.925–1.88)	0.127	1.28(.896–1.832)	0.174
ICU transfer^¥^	1.03(1.002–1.06)	0.038	1.04(1.01–1.08)	0.02	1.03(0.936–1.14)	0.531	1.02(0.925–1.13)	0.664
Ventilatory support (invasive/non invasive)^¥^	1.04(1.01–1.07)	0.012	1.05(1.02–1.085)	0.005	1.04(0.939–1.15)	0.473	1.02(.918–1.126)	0.747

*Results obtained from Cox regression. ^¥^ results obtained from logistic regression. Adjusted associations are controlled for the effect of age, gender, cardiovascular disease and admission creatinine.

### Haemodynamic parameters

Both systolic and diastolic blood pressure were found significantly and directly associated to all-cause mortality (HR = 0.986 per mmHg increase in systolic blood pressure, 95% CI 0.974–0.998, *P* = 0.023 and HR = 0.977 per mmHg increase in diastolic blood pressure, 95%CI 0.959–0.996, *P* = 0.017 ), while no association was observed with the heart rate (HR = 1.009 per bpm increase in heart rate, 95%CI 0.997–1.020, *P* = 0.129). The prognostic association between hsTnT or D-dimer and mortality remained highly significant also after adjustment for both these haemodynamic parameters, as shown in Table 4.

**Table 4:** Unadjusted and blood pressure adjusted associations of 1 SD increase of D-dimer and hsTnT with all-cause mortality.

	Unadjusted		Adjusted for SBP		Adjusted for DBP	
	HR/OR (95%CI)	*P*-value	HR/OR (95%CI)	*P*-value	HR/OR (95%CI)	*P*-value
D-dimer	1.09 (1.03–1.15)	0.001	1.48 (1.11–1.98)	0.008	1.73 (1.19–2.51)	0.004
hsTnT	1.20 (1.07–1.34)	0.002	1.15 (1.03–1.30)	0.016	1.16 (1.04–1.31)	0.010

### Determinants of hsTnT levels

The Table 5 shows the characteristics of the population stratified by the median value of hsTnT. Comorbidities including diabetes mellitus, chronic obstructive pulmonary disease, arterial hypertension, prior history of CVD and cerebrovascular disease, dementia, chronic kidney disease were more prevalent in patients with higher hsTnT levels. They also had increased levels of D-dimer (*P* = 0.021) and CRP (*P* < 0.001) and lower PaO_2_ (*P* < 0.001) and PaO_2_/FiO_2_ ratio (*P* = 0.001).

**Table 5:** Differences in the clinical characteristics of the population according to median levels of admission hsTnT.

Variable	N	Admission hs Troponin		*P*-Value
		Below the median	Above the median	
Age, years	200	59 (13)	78 (11)	< 0.001
Male sex, n (%)	200	39 (38)	30 (31)	0.302
Current smoking, %	187	4 (4)	2 (2)	0.494
PaO_2_*	182	77 (63–87)	61 (50–74)	< 0.001
PaO_2_/FiO_2_*	194	300 (240–371)	257 (195–324)	0.001
Glucose, mg/dL	194	121 (34)	148 (72)	< 0.001
Haemoglobin, g/dl*	195	14 (12.7–14.9)	12.9 (11.4–14.6)	0.006
Leukocyte count, cells/μl*	197	6380(4780–8330)	6690(5240–10,060)	0.079
Lymphocyte count, cells/μl*	197	1100 (690–1330)	835 (540–1116)	< 0.001
Neutrophils count, cells/μl*	194	4500 (3120–6240)	5600 (3447–7665)	0.036
Platelets count, cells/μl*	197	196 (149–248)	175 (129–238)	0.126
INR*	193	1.21 (1.13–1.28)	1.25 (1.18–1.48)	0.002
Total Bilirubin, mg/dL*	194	0.44 (0.32–0.63)	0.56 (0.38–0.76)	0.038
Aspartate transaminase, U/L*	186	32 (24–50)	34 (25–51)	0.737
Alanine transaminase, U/L*	195	33 (23–49)	22 (14–36)	< 0.001
Creatinine, mg/dL*	195	0.89 (0.78–1.03)	1.24 (0.96–1.83)	< 0.001
Sodium, mEq/L*	196	138 (135–140)	138(134–141)	0.467
Potassium, mEq/L*	192	3.98 (3.73–4.25)	4.13 (3.84–4.47)	0.007
D-dimer, mg/L*	75	0.32 (0.21–0.69)	0.67 (0.35–1.10)	0.021
Lactate Dehydrogenase, U/L*	109	283 (216–405)	357 (251–467)	0.039
Creatine phosphokinase, U/L*	100	76 (50–213)	92 (56–207)	0.587
High sensitivity troponin T, ng/L*	200	9 (6–12)	41 (26–85)	< 0.001
Procalcitonin, ng/mL*	177	0.08 (0.05–0.16)	0.19 (0.10–0.65)	< 0.001
C-reactive protein, mg/dl*	193	4.31 (1.79–10.67)	8.88 (4.87–18.49)	< 0.001
**Comorbidities**				
Chronic obstructive pulmonary disease, n (%)	200	4 (4)	23 (24)	< 0.001
Asthma, n (%)	199	8 (8)	4 (4)	0.286
Diabetes mellitus, n (%)	200	9 (9)	29 (30)	< 0.001
Arterial hypertension, n (%)	200	31 (30)	63 (65)	< 0.001
History of cardiovascular disease, n (%)	200	18 (17)	49 (50)	< 0.001
History of cerebrovascular disease, n (%)	200	3 (3)	18 (19)	< 0.001
Diagnosis of dementia, n (%)	200	2 (2)	13 (13)	0.002
Diagnosis of Cancer, n (%)	200	12 (12)	17 (17)	0.238
Haematological malignancy, n (%)	200	0 (0)	4 (4)	0.037
Chronic kidney disease, n (%)	200	2 (2)	14 (14)	0.001
Dialysis, n (%)	200	0 (0)	3 (3)	0.072
Chronic liver disease, n (%)	200	1 (1)	6 (6)	0.045

*P*-values are derived from independent samples Student’s T Test or the Mann Whitney test for continuous variables and the chi squared test for categorical variables; *Median and interquartile range.

In linear regression models, including PaO_2_, D-dimer and CRP as independent variables further adjusted for history of CVD, creatinine, sex and age, D-dimer and CRP presented a stronger association with the levels of hsTnT than PaO2 (Table 6). Linear mixed model analysis including the subgroup of patients with available serial measurements of hsTnT, D-dimer and CRP, changes in D-Dimer followed the fluctuations in hsTnT concentrations after controlling for age, gender, creatinine and history of CVD (coefficient = 0.014, 95% CI 0.01–0.024, *P* = 0.003). A weaker association was also detected between changes in CRP and changes of hsTnT concentrations after adjustment (coefficient = 0.053, 95% CI 0.0173–0.089, *P* = 0.004).

**Table 6:** Determinants of admission hsTnT levels by linear regression analysis.

Variable	Univariable		Multivariable	
	*Standardized Coefficient	*P*-value	*Standardized Coefficient	*P*-value
Age	0.607	< 0.001	0.395	0.002
Sex	− 0.048	0.500	− 0.105	0.327
History of Cardiovascular disease	0.319	< 0.001	0.130	0.242
Admission creatinine	0.321	< 0.001	0.217	0.04
Admission CRP	0.338	< 0.001	0.195	0.226
Admission PaO2	− 0.179	0.016	0.015	0.890
Admission D-Dimer (n = 84)	0.303	0.008	0.238	0.028

*Coefficients correspond to 1-unit change in natural log transformed hs Troponin.

### Attenuation of the risk related to high hsTnT levels by COVID-19 treatment

**Figure 2:**
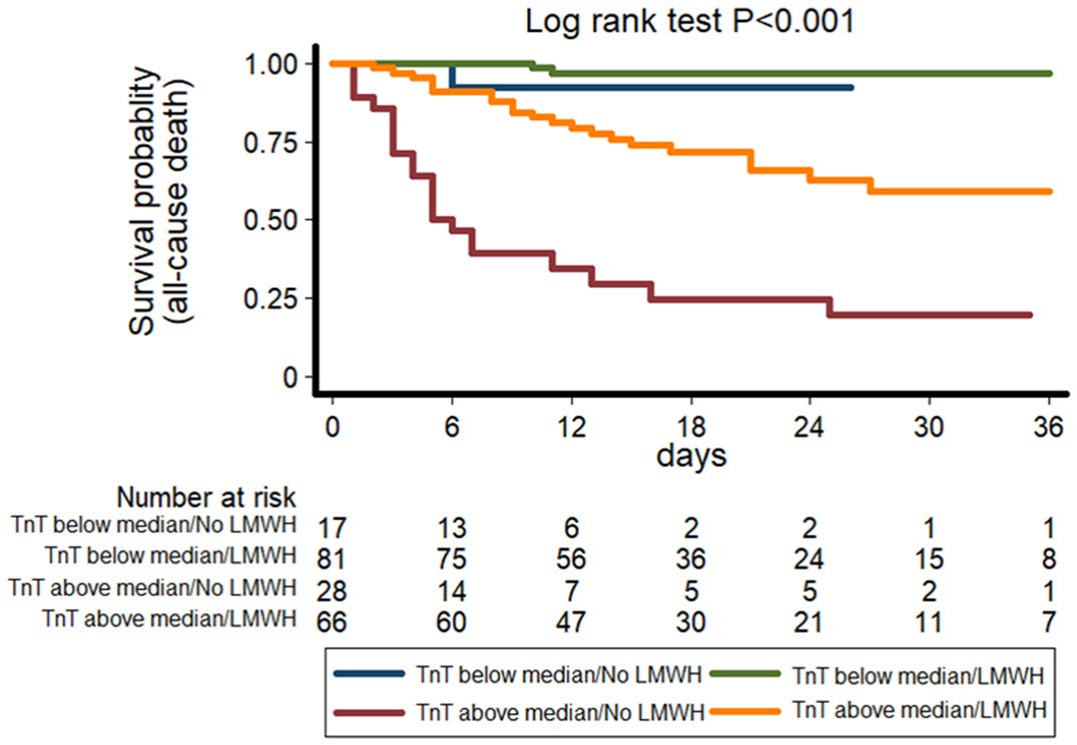
Kaplan–Meier survival curves for all-cause death according to hsTnT levels above and below median and the presence or absence of heparin treatment during hospitalization (green line = TnT below median, heparin; blue line = TnT below median, no heparin; orange line = TnT above median, heparin; red line = TnT above median, no heparin). *TnT: Troponin T; LMWH: Low Molecular Weight Heparin.*

The prognostic role of hsTnT at admission remained significant also after adjustment for the main drugs used for the treatment of COVID-19, including low molecular weight heparin (LMWH), hydroxychloroquine, antibiotic, immunomodulating drugs (baricitinib and tocilizumab) and corticosteroids. In multivariable Cox regression models including the same drugs and controlling for age, sex, history of CVD, creatinine and hsTnT, only LMWH remained significantly associated with mortality after bootstrapping (HR = 0.271, 95% CI 0.140–0.525, *P* < 0.001). Thus, we tested whether the risk of mortality related to high hsTnT level was somewhat attenuated by the treatment with heparin. When added to the model testing the relationship between hsTnT levels and mortality, the interaction between hsTnT and use/non-use of heparin during hospitalization was highly significant (HR = 0.424, 95% CI 0.239–0.752, *P* = 0.003), suggesting that heparin could modify the relationship between hsTnT levels and mortality risk. Indeed, in the group of subjects with levels of hsTnT above the median values the treatment with heparin significantly lowered the risk of mortality compared to untreated subjects (HR = 0.294, 95% CI 0.152–0.569, *P* < 0.001), while the same difference was not detected in subjects with hsTnT lower than the median (HR = 0.06, 95% CI 0.002–2.100, *P* = 0.121) (Fig. 2).

## Discussion

In this single-centre, prospective, observational study we report that: hsTnT five-fold elevation is the greatest difference observed among a large plethora of clinical and biochemical parameters characterizing baseline phenotype of 266 patients hospitalized for COVID-19 and stratified for mortality; baseline hsTnT and D-dimer, but not CRP, are associated with in-hospital COVID-19 mortality; D-dimer accounts for a substantial proportion of the hsTnT variability; anticoagulant treatment during hospitalization substantially reduced the mortality risk, a benefit particularly evident in subjects with elevated hsTnT.

Our study aimed at exploiting, from a clinical in-hospital point of view, the complex interplay between inflammation, coagulation and cardiac injury. We showed that the activation of inflammatory/coagulation pathways might explain a large proportion of the increase in hsTnT levels commonly observed in the COVID-19, probably higher than the severity of hypoxia. Although changes in hsTnT levels during hospitalization were significantly related to the fluctuations of the CRP and D-dimer concentrations, only D-dimer presented an association with mortality similar to hsTnT. These findings suggest that cardiac injury and coagulation pathways might be relevant in defining the risk of mortality related to elevated hsTnT levels in patients with SARS-CoV-2-related pneumonia.

In our observation we found hsTnT, among a large numbers of serum biomarkers, the parameter with the major difference at baseline between survived vs deceased patients hospitalized for COVID-19. Cardiac injury, marked by troponin (both T^15^ and I^16^) elevation, is frequent in patients with COVID-19 and related with adverse outcomes^17,18^. In fact, while the most common clinical presentation of the SARS-CoV-2 infection is bilateral interstitial pneumonia often associated to respiratory failure, several reports have supported the capacity of cardiac injury, indirectly assessed by cardiac troponin due to the in-hospital real-life setting, to inform on the mortality risk in COVID-19^1–3,17^. Troponin elevation has been related with a large set of inflammatory serum markers, such as CRP, ferritin, IL-6, tumour necrosis factor-α and also thrombotic markers such as D-dimer. No previous studies^17^, however, have accurately explored the potential and complex interaction between these three biologic systems and thus defined the putative determinants of hsTnT elevation levels in COVID-19.

In accordance with current literature, we document that hsTnT is associated to an increased risk of in-hospital mortality in subjects with and without previous history of CVD^17^, suggesting that the exclusive presence of coronary atherosclerosis is unlikely to completely explain the high mortality risk of these patients^2^. One plausible hypothesis is that cytokine storm induced by the SARS-CoV-2-related pneumonia can promote the activation of the coagulation system which, in turn, can result in a systemic microangiopathy, multi-organ failure and increased mortality^19^. However, the relevance of the inflammation in defining the mortality outcome has recently been debated, as observed by Sinha et al.^20^. Indeed, similar to hsTnT, an increased D-dimer concentrations have been related to the mortality risk of infected patients ^3,7^.

In our observation, also, we found that CRP, beyond its association with hsTnT, was related to the risk of ICU admission and requirement of mechanical ventilation, both outcomes defining the severity of the pneumonia. However, CRP was not predictive of mortality. Instead, D-dimer and hsTnT presented a strong relationship with mortality, but they were not informative on the risk of respiratory complications eventually leading to ICU admission. It’s plausible that while CRP might reflect more closely the pulmonary stage of the disease and the severity of cytokine storm related to pneumonia^21^, the elevation of D-dimer and hsTnT might sign the evolution towards a systemic involvement of the infection. This is in line with the fact that, in our study, we observe a larger variability of hsTnT explained through D-dimer levels instead that through the severity of the hypoxia related to pneumonia.

In our exploratory analysis we found that also CRP might explain hsTnT variability. This is consistent with several observations which described how both coagulation and inflammation are involved in hsTnT elevation^22^. However, in our study hsTnT association with CRP was weaker than with D-dimer. This and the fact that only D-dimer, and not CRP, was associated to an increase in all-cause mortality, strongly support the predominant relevance of coagulopathy in the natural history of the systemic SARS-CoV-2 infection^23^. The development of disseminated intravascular coagulation (DIC) in patients with more severe interstitial pneumonia could partially explain the stronger relationship of D-dimer and hsTnT with the mortality risk compared to CRP. However, in ours and other studies, the pattern of coagulopathy observed during SARS-CoV-2 infection was substantially different to the DIC commonly seen in sepsis^24^. Indeed, our population did not show severe thrombocytopenia which usually accompanies the sepsis-related DIC, and the D-dimer concentrations often reached higher levels than those expected in DIC. It has been repeatedly emphasized that most patients with COVID-19 related coagulopathy would not be classified as having DIC according to the recommendations of the International Society on Thrombosis and Haemostasis^7,24^. Rather, the activation of the coagulation system observed during COVID-19 and its association with multiple organ dysfunction might reflect the generalised state of endothelitis which accompanies the SARS-CoV2 infection. Independently from its origins, the presence of a coagulopathy in COVID-19 is undebatable: in *post-mortem* studies, a microangiopathy characterized by typical microvascular platelet-rich thrombotic depositions has been described not only in small vessels of the lung parenchyma but also in other tissues, such as the myocardium^8^. Such microangiopathy could account more directly for the multi-organ failure and death in COVID-19 patients. Following this interpretation, the elevation of hsTnT might represent also an early marker of a systemic macroangiopathic involvement during COVID-19 infection, and its high prognostic capacity might depend on its higher sensitivity and specificity compared to other markers of tissue injury^4^.

In our study only the treatment with low molecular weight heparin showed convincing evidence for an attenuation of the mortality risk. This is in line with the current literature: observations about anticoagulation therapy show promising results and expert opinions have recommended the use of prophylactic low molecular weight heparin in patients with COVID-19 in absence of clinical contraindications^6,25^. Ayerbe et al. reported an association between heparin and lower mortality in 2075 patients admitted with COVID-19 in 17 hospitals in Spain^9^. In a retrospective study including 449 patients with severe COVID-19 in China, 99 of which received heparin, 28-day mortality of heparin users was lower than that of non-users in patients with D-dimer levels more than six-fold the upper limit of normal^7^. An observational study including 2,773 patients hospitalized with laboratory-confirmed COVID-19 within the Mount Sinai Health System in New York City showed that systemic anticoagulation was associated with improved outcomes^6^. We recently found that low molecular weight heparin was the only therapeutic factor independently associated with survival in a propensity-score adjusted analysis on 315 patients with COVID-19^10^. As for other systemic diseases with high prevalence in older population, the prescription of an anticoagulation treatment should be weighed against the risk of bleeding. Otherwise, this may lead to controversial results. Indeed, Pesavento et al. showed that, in a cohort of 324 patients showed that (sub)therapeutic doses of anticoagulants, when compared to standard prophylactic ones, are related to a major risk of bleeding (HR 3.89; 95%CI, 1.90 to 7.97) despite no beneficial effect on overall mortality^26^.

In our study, we report, for the first time, that the benefit from anticoagulation therapy was more evident in subjects with higher hsTnT elevation. The positive effects obtained from the prescription of low molecular weight heparin and particularly in patients with higher hsTnT levels confirm the contribution of systemic coagulopathy to the myocardial injury detected in COVID-19. Moreover, due to the bleeding risk implied with the LMWH treatment, the identification of parameters that enable selection of patients who could receive the greatest benefits from anticoagulation therapies might be crucial to optimize the risk–benefit balance related to this treatment. Our data suggest that a prophylactic dose of low molecular weight heparin during admission might be particularly indicated in patients presenting with mild to moderate elevation of hsTnT levels. If these patients might benefit from the use of higher dose thromboprophylaxis than is generally given remains unknown. However, ongoing multicentre, randomized, controlled trials will be able to address this question (NCT04372589, NCT04367831, NCT04345848, and NCT04366960).

Our study has some strengths and limitations. The availability of makers informing on the severity of hypoxia, inflammatory response and coagulation abnormalities provided us with the opportunity to explore their relative importance in causing an increase in the hsTnT level and its association with mortality. In a subset of patients, serial measures of hsTnT, CRP and D-dimer were also available, making it possible, for the first time, to relate changes in hsTnT levels with the fluctuation of the inflammatory and coagulation responses to the disease. The presence of several outcome measurements enabled the assessment of the relative importance of the different pathways explored in the study with multiple complications of patients with COVID-19 infection. However, our study is monocentric, thus the results might be affected by local practice in the management of the COVID-19 infection and not be generalizable to other health care settings. Also, we used only D-dimer and CRP as markers of coagulation abnormalities and inflammation. Furthermore, given the pressure imposed by the elevated number of admissions in a short timeframe, we were unable to provide a better characterization of the myocardial injury with other, well-established circulating markers (such as cardiac natriuretic peptides) or with imaging studies (including echocardiography). This has also precluded the opportunity to explore which proportion of the described changes in the marker of cardiac injury could be attributed to pulmonary haemodynamic changes and their potential relationships with the inflammatory and coagulation abnormalities reported in our population. For the same reasons, we were not able to obtain a more accurate assessment of the COVID-19-related coagulopathy and inflammatory response. However, previous report have shown that the elevation in the circulating levels of D-dimer is amongst the most typical characteristics of the coagulopathy characterizing the SARS-CoV-2 infection and its assessment is commonly used in clinical practice as a screening tool for the presence of trombo-embolic events in other diseases^27^. Similarly, CRP is commonly used in clinical practice to monitor the severity of the inflammatory response resulting from acute infections and is also considered a good marker to assess the inflammatory-related risk of cardiovascular events^28^. The analysis on the beneficial effects of treatments should be interpreted cautiously, as it was not conducted on randomized groups and might be therefore affected by several measured and unmeasured confounding factors. Furthermore, we were not able to recover clear information on the indication for anticoagulation treatment.

In summary, our data support the hypothesis that the increase of hsTnT levels detected in COVID-19 infection is multifactorial, with inflammation and coagulation responses representing important factors contributing to this clinical manifestation. CRP can be used to monitor inflammation but might not represent the most accurate marker to discriminate between the severity of the respiratory and systemic inflammatory involvement during SARS-CoV-2-related pneumonia. Instead, an elevation of the D-dimer might sign the evolution towards a systemic involvement of the infection, with the development of a coagulopathy that could lead to multiorgan failure. Low molecular weight heparin is, indeed, a promising treatment to counteract the risk of mortality related to systemic COVID-19. Further studies are needed to clearly define the pathogenesis of COVID-19 and provide useful insight for tailored therapies. Troponin, in a context where risk stratification is of primary importance, due to its ability to predict mortality and the relevance of if prognostic capacity even in terms of identification of patients who would benefit more from specific treatments (such as anticoagulation), might play a fundamental role.

## Data Availability

Data are available upon reasonable request.

## Abbreviations

COVID-19: Coronavirus Disease 2019
CRP: C-reactive protein
CVD: Cardiovascular disease
hsTnT: High-sensitivity troponin T
SARS-CoV-2: Severe Acute Respiratory Syndrome—Coronavirus – 2
LMWH: Light Molecular Weight Heparin
